# Greener Solvent-Based Processing of Magnetoelectric Nanocomposites

**DOI:** 10.1021/acssuschemeng.1c06967

**Published:** 2022-03-22

**Authors:** A. C. Lima, N. Pereira, C. Ribeiro, S. Lanceros-Mendez, P. Martins

**Affiliations:** † Physics Centre of Minho and Porto Universities (CF-UM-UP), Universidade do Minho, 4710-057 Braga, Portugal; ‡ INL-International Iberian Nanotechnology Laboratory, 4715-330 Braga, Portugal; ∥ CEB-Centre of Biological Engineering, University of Minho, 4710-057 Braga, Portugal; ⊥ BCMaterials, Basque Center for Materials, Applications and Nanostructures, UPV/EHU Science Park, 48940 Leioa, Spain; # IKERBASQUE, Basque Foundation for Science, 48009 Bilbao, Spain; ∇ IB-S Institute of Science and Innovation for Sustainability, Universidade do Minho, 4710-057 Braga, Portugal

**Keywords:** green manufacturing and engineering, nanoscience and nanotechnology, green solvents, sustainable materials, magnetoelectric

## Abstract

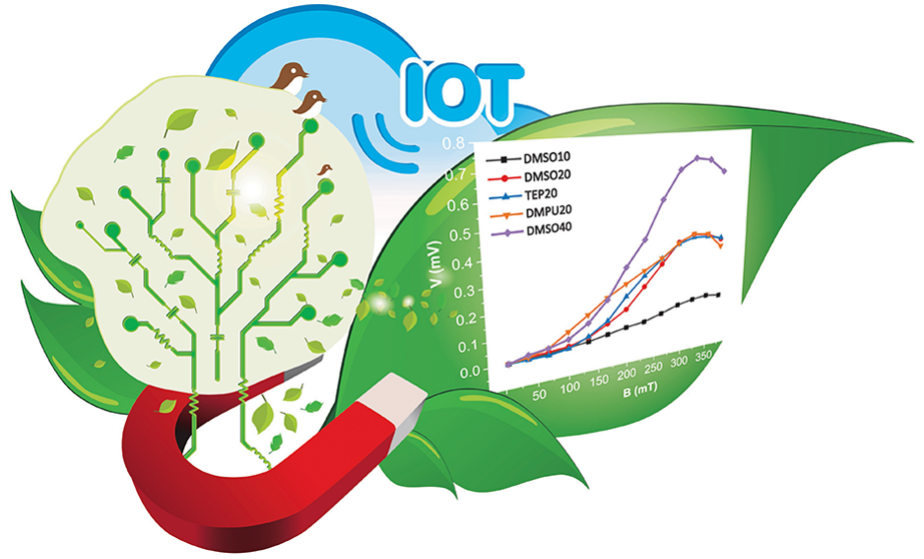

Polymer-based magnetoelectric (ME) nanocomposites are an enabling material technology for a wide range of applications in the area of digitalization strategies. Due to its highest piezoelectric response among polymers, poly(vinylidene fluoride-trifluoroethylene) (PVDF-TrFE) is the piezoelectric matrix most used in polymer-based ME materials with over 80% of the total reports, with the resulting composites typically processed from solutions with *N*,*N*-dimethylformamide (DMF), a toxic solvent. Nevertheless, environmentally friendlier approaches and sustainable technologies are increasingly being required. This work demonstrates that P(VDF-TrFE)/Co_2_Fe_2_O_4_ nanocomposites can be successfully prepared from solution using three different environmentally friendlier solvents: dimethyl sulfoxide (DMSO), *N*,*N*′-dimethylpropyleneurea (DMPU), and triethyl phosphate (TEP) with different dipole moments. It is shown that the prepared composite films, with a maximum ME voltage coefficient of 35 mV cm^–1^ Oe^–1^ and a maximum sensitivity of 2.2 mV T^–1^, are suitable for applications, highlighting the path for a new generation of more sustainable ME sensors.

## Abstract 2

The state-of-the-art manufacturing process of polymer-based ME materials uses toxic organic solvents. Through an optimized thermodynamic procedure such hazardous materials may be replaced by green solvents.

## Introduction

The link between the physical world and the Internet has been a driving force in enhancing peopleʼs quality of life, which has culminated in the Internet of Things (IoT) concept.^1−3^ This technology allows a more effective and ubiquitous interrelation between humans and electronic devices, modifying the paradigm of the user’s interactions. In such human–machine connectivity, the user, who previously interacted with a physical object in a voluntary way, starts to interact with the object simply by using it: the “I-user”.^4,5^ In addition, the rapid evolution of the digitalization wave, leading to the interconnection in real time of every possible “Things” (home appliances, mobile phones, cars, or smart houses, among others), demands also a new generation of materials^6^ that allows lightness, flexibility, and improved integration at reasonable prices.^4,7^ Thus, a new generation of materials called “smart materials” are being developed to be integrated in a variety of objects and devices with the objective to sense temperature, pressure, impact, deformation, and other relevant parameters.^8−10^ Additionally, some of those types of materials can also act as energy harvesters^11,12^ or actuators.^13,14^

Magnetoactive smart materials’ contribution to the IoT is particularly relevant since they will trigger the fabrication of antennas; bioelectronics devices; proximity, magnetic, and current sensors; and actuators, among others.^5,15^ Magnetoactive materials can be produced by combining magnetic fillers and polymeric/ceramic matrices, and in such systems, the application of an external AC/DC magnetic field triggers a physical change in the composite.^16^ Among those materials, magnetoelectric (ME) structures are among the most exciting ones, once they allow an efficient coupling between the electrical and magnetic orders of the matter.^17^ In addition, ME materials permit the development of novel and conventional applications, the latter based on new approaches, such as in the case of magnetic sensors, that match the current requirements of the IoT-related industries in terms of cost, size, flexibility, detection limits, and noise.^18,19^ ME materials can also solve problems related to the powering of conventional sensors (periodic replacement, capacity limitations, and maintenance costs) due to their aptitude to harvest surrounding energy from harsh/inaccessible locations, exhibiting the same harvesting benefits of piezoelectric materials but with improved power output and higher mechanical stress.^20^

ME composites, with ME coupling coefficients ≈1000 times higher than single-phase MEs, are usually categorized according to the connectivity of the two components into 0–3 nanocomposites and 2–2 laminated composites.^21,22^ Laminated composites have been largely explored based on their superior ME coefficient (≈some V cm^–1^ Oe^–1^), though the stress transfer of the coupling layer between piezoelectric and magnetostrictive layers brings undesired relaxation and electrical loss problems.^23^

Nanocomposites despite exhibiting a lower ME coupling coefficient (≈some mV cm^–1^ Oe^–1^) do not exhibit the abovementioned performance limitations, once the magnetostrictive nanoparticles are equally dispersed in the piezoelectric matrix.^24^ Polymer-based ME nanocomposites also offer optimized features with respect to low-temperature processing, compatibility with additive manufacturing technologies, and tunable mechanical features for flexible and/or large area devices,^25,26^ thus permitting the manufacture of products that are more eco-friendlier and safer.^23^

Due to their highest dielectric constant and electroactive response, including the piezoelectric (|*d*_33_| ≈ 30 pC N^–1^) effect, and the capability to crystallize directly in the β-phase (the phase that displays optimized ferroelectric, pyroelectric, and piezoelectric properties), poly(vinylidene fluoride-trifluoroethylene), P(VDF-TrFE), with molar ratios of vinylidene fluoride (VDF) between 50 and 80%, is the copolymer preferentially used (≈80% of the total published papers^23,27^) for the fabrication of ME nanocomposites.

For most applications, P(VDF-TrFE) is processed from the melt, such as by extrusion or injection molding, where the material is converted from a liquid melt into a solid form with a defined structure and shape,^28,29^ with melt extrusion being a common method to produce oriented flexible chain polymers.^30,31^ Nevertheless, in the growing area of printed electronics and advanced coatings, increasingly requiring sensors and/or actuators, piezoelectric materials in general and P(VDF-TrFE) in particular need to be processed from solutions,^32^ which requires in some cases a specific postprocessing treatment.^24^ The most utilized printing/coating techniques include screen printing or doctor blade.^17,23^ In the field of printed electronics, P(VDF-TrFE) is mostly prepared after dissolution in *N*,*N*-dimethylformamide (DMF), methyl ethyl ketone (MEK), tetrahydrofuran (THF), or *N*-methyl-2-pyrrolidone (NMP), and all of them are hazardous, toxic, and dangerous to be used at a large scale and, consequently, should be avoided.^27,33^

In this way, one of the key enabling challenges in printable polymer-based MEs is to change the abovementioned solvents by less hazardous and environmentally friendlier ones, the so-called “green” solvents, aiming to decrease the environmental footprint of the ME materials.^23,34^ The choice of solvent must also take into account its dipole moment once such a value significantly influences its polarizability which in turn affects the piezoelectricity, pyroelectricity, triboelectricity, crystallinity, and dipole alignment capability of the polymer that is obtained from solutions.^35,36^

**Figure 1:**
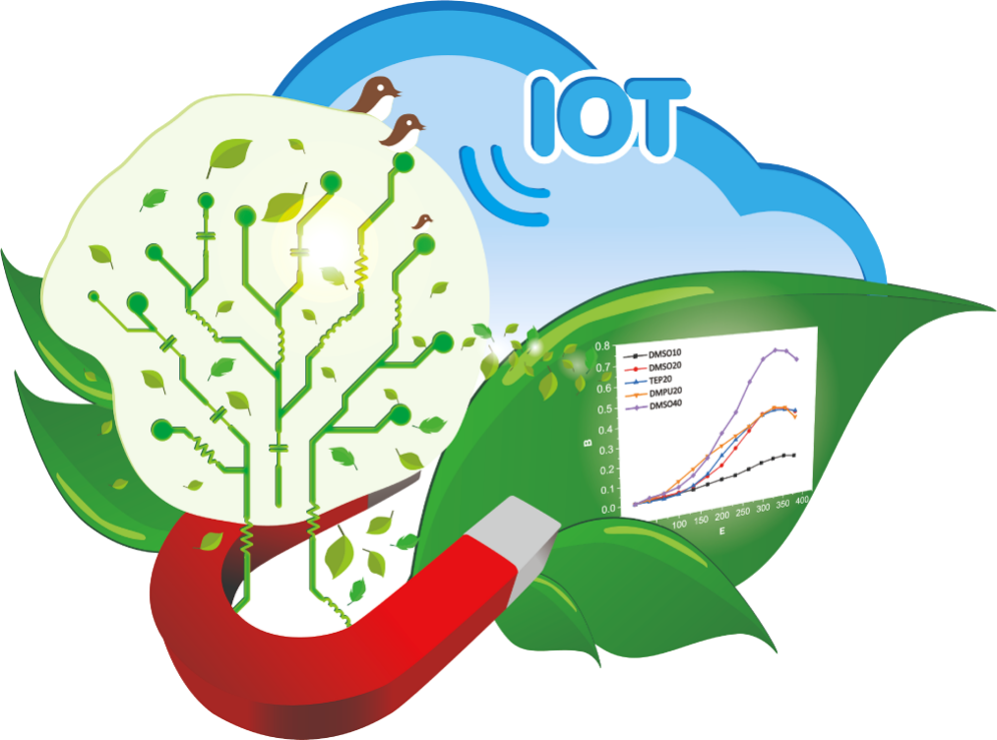
Scheme of the main issues addressed in this work: environmentally friendly approaches regarding the development of magnetoelectric smart materials for IoT-related applications.

Thus, in this work, P(VDF-TrFE)/CoFe_2_O_4_ ME nanocomposites have been developed using three different green solvents, dimethyl sulfoxide (DMSO), *N*,*N*′-dimethylpropyleneurea (DMPU), and triethyl phosphate (TEP), aiming at sensing applications (Figure 1). Further, different dipole moments of the solvents also allow assessment of the solvent–polymer interactions, allowing the improvement of final material properties.

Regarding the magnetostrictive phase and knowing that minable deposits of heavy rare-earth elements, such as dysprosium (Dy), terbium (Tb), or neodymium (Nd), are heterogeneously and sparsely distributed over the planet, thus being important to diminish the quantities used to solve supply issues, material cost limitations, and environmental problems,^37,38^ those materials should be suppressed in the developed ME nanocomposites. Thus, CoFe_2_O_4_ will be used once it, along with its hysteresis (coercivity of ≈0.25 T), high magnetostrictive coefficient (up to ≈220 ppm), and chemical stability, can be produced through eco-friendly green methods.^39−42^

## Experimental Section

### Materials

CoFe_2_O_4_ (CFO) nanoparticles of 35–55 nm were acquired from Nanostructured & Amorphous Materials, Inc. (USA). Dimethyl sulfoxide (DMSO), *N*,*N*′-dimethylpropyleneurea (DMPU), and triethyl phosphate (TEP) were obtained from Merck (Germany), LaborSpirit (Portugal), and Sigma-Aldrich (USA), respectively. P(VDF-TrFE) (Solvene 250, VDF/TrFE = 70/30) was provided by Solvay (Brussels, Belgium). All of the chemicals, polymers, and magnetic nanoparticles were utilized as received from the suppliers. The characteristics of each solvent can be found in Table 1.

**Table 1:** Relevant Properties of Different Solvents Used to Dissolve P(VDF-TrFE)^43−46^

	DMF	DMSO	DMPU	TEP
dipole moment (D)	3.90	3.96	4.17	2.86
boiling point (° C)	153	189	247	215
hazard statement^a^	highly flammable liquid and vapor	combustible, but will not ignite readily	flammable liquid and vapor	nonflammable
	harmful in contact with skin or if inhaled	slightly irritant to eyes, but not relevant	eye contact may result in permanent eye damage	harmful if swallowed
	causes serious eye irritation	no effects known after inhalation	harmful if swallowed	causes serious eye irritation
environmental impact/usability^b^	high/undesirable use	some/usable	some/usable	none:/recommended

a According to Regulation (EC) No. 1272/2008.

b According to the CHEM21 method and GSK’s solvent selection guide.^47−50^

### Processing

Nanocomposite films with thickness around 40–60 μm were prepared by adding the desired amount of CFO to DMSO, DMPU, or TEP solvent and later subjected (5 h) to ultrasound treatment (ATU ATM 3LCD) to ensure that CFO was well dispersed in the solvent and to prevent aggregation.^51^ Next, the polymer in the powder form was added, and the resulting blend was mixed using a Teflon mechanical stirrer (Heidolph instruments, Schwabach, Germany) until the complete dissolution of P(VDF-TrFE). Flexible multiferroic films were obtained by spreading (at room temperature) the mixture on a very clean glass substrate. P(VDF-TrFE) crystallization and solvent evaporation were achieved in a postprocessing step by maintaining the samples inside a forced convection oven (JP Selecta 2005165) for (i) 30 min at 230 °C for DMPU films, (ii) 30 min at 165 °C for DMSO films, and (iii) 30 min at 180 °C for TEP films. Each solvent evaporation temperature was selected taking into account the following premises: (i) temperature should be lower (≈10%) than the solvent’s boiling point (Table 1) to allow slow solvent evaporation, leading to nonporous compact films and (ii) temperature should be the lowest promoting a compact, homogeneous, and nonbrittle film.

After the specific/optimized time in the oven, the films were cooled to room temperature under ambient atmosphere. Two sets of samples were prepared, one with 20 wt % CFO and prepared with different solvents, allowing us to understand the effect of the used solvent on the ME performance, and the other with varying CFO wt % (0, 10, 20 and 40 wt %) in the same solvent (DMSO) to understand the effect of increasing filler content (Table 2).

**Table 2:** P(VDF-TrFE)-Based Composite Samples and Respective Nomenclature

	CoFe_2_O_4_ (wt %)			
solvent	0	10	20	40
DMSO	P@DMSO	P@DMSO/CFO10	P@DMSO/CFO20	P@DMSO/CFO40
DMPU	X	X	P@DMPU/CFO20	X
TEP	X	X	P@DMSO/TEP20	X

### Characterization

Sample morphology was evaluated by scanning electron microscopy (SEM, NanoSEM, FEI Nova 200) using an accelerating voltage of 10 kV. Previously, all samples were coated (using a sputter coating Polaron, model SC502) with a very thin (≈30 nm) conductive Au layer. Sample thickness was calculated using ImageJ software on SEM images (5 measurements for each sample).

The wt % of the polymer at the polymer–CFO interface (*m*_I_) was determined using eq 1
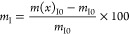
1where *m*_I0_ is the mass of pristine P(VDF-TrFE) at the temperature at which the mass loss rate is maximum and m(*x*)_I0_ is the mass of the composite containing a given wt % of CFO nanoparticles that has not degraded at the temperature at which the mass loss rate of pristine P(VDF-TrFE) is maximum.^52^

Differential scanning calorimetry (DSC) was performed using a PerkinElmer DSC 6000 under a flowing N_2_ atmosphere from 25 to 200 °C (with a 10 °C min^–1^ heating rate). The samples were placed in aluminum pans (≈50 μL) with perforated lids to permit the release/removal of volatiles. The degree of crystallinity (χ_c_) of the samples was determined using eq 2
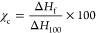
2where Δ*H*_f_ is the melting enthalpy and Δ*H*_100_ is the melting enthalpy for a 100% crystalline sample (103.4 J g^–1^).^53^

Thermogravimetric analysis (TGA) with a heating rate of 10 °C min^–1^ was executed on a Q600 TGA TA thermobalance from 40 to 800 °C under a N_2_ atmosphere (flow of 50 mL min^–1^). Samples (≈10 mg) were previously placed in open ceramic crucibles.

The room-temperature stress–strain measurements in tensile mode at a deformation rate of 0.5 mm min^–1^ were performed using an Autograph AG-IS (Shimadzu) 500 N to assess the mechanical properties of samples. The elastic modulus, *Y*, was calculated in the linear regime of the stress–strain curves after Hookeʼs law.
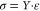
3For both dielectric and electrical conductivity experiments, circular gold electrodes (5 mm diameter) were deposited onto both sides of the films on a Polaron model SC502 sputter coater.

The capacity (*C*) and the dielectric losses (tan(δ)) of the films were evaluated at room temperature from 1 Hz to 1 MHz and with an applied voltage of 0.5 V in a QuadTech 1920 apparatus. Gold electrodes (5 mm diameter) were vacuum-evaporated on both sides of each sample. Then, the real part of the dielectric permittivity (ε′) was obtained using the parallel plate capacitor approximation considering the geometry and dimension of each sample (thickness *t* and area *A*) using eq 4.
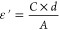
4The DC volume electrical conductivity of the films was obtained by assessing the room-temperature characteristic IV curves with a Keithley 6487 picoammeter and voltage source. The electrical resistivity (ρ) was then calculated taking into account the geometrical parameters according to
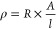
5where *R* is the film resistance calculated by the inverse of the slope of the *I*(*V*) function, *t* is the thickness, and *A* is the area of the electrodes. The electrical conductivity (σ) is calculated as the inverse of the resistivity.

For the assessment of the *d*_33_ piezoelectric coefficient, the samples were first poled by a corona discharge, after a previously optimized procedure (temperature of 120 °C, inside a homemade chamber, 10 kV applied voltage, ≈2 cm distance between the sample and tip, 1 h poling time, and cooling to room temperature under an applied electric field). Then, the piezoelectric *d*_33_ coefficients were measured with a *d*_33_ meter (model 8000, APC int, Ltd.).

To obtain the out-of-plane ME coefficient (α_33_), DC (bias) and AC magnetic fields (*H*_AC_) were applied simultaneously along the same direction of the electric polarization of the P(VDF-TrFE) composite film, that is, perpendicular to the surface. The α_33_ was calculated using eq 6.
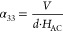
6The AC driving magnetic field (with a maximum of 3.98 Oe) was provided by a pair of Helmholtz coils. The external bias field was provided by an electromagnet with a maximum value of 1.2 T. The induced ME voltage (*V*) in the samples was measured using a Stanford Research Lock-in amplifier.

The sensitivity (*S*_DC_) of the ME composite was determined using eq 7.
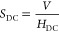
7where *H*_DC_ is the magnitude of the DC magnetic field.

## Results and Discussion

### Morphological Features

**Figure 2:**
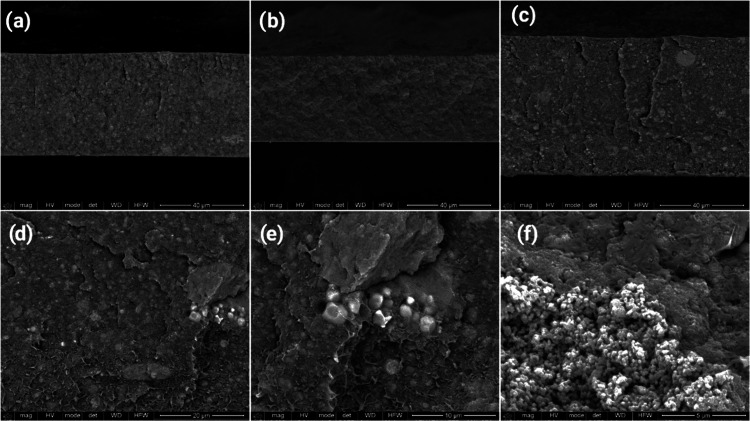
Representative SEM images of (a) P@DMPU/CFO20, (b) P@DMSO/CFO20, and (c) P@TEP/CFO20. SEM images of P@TEP/CFO20 at different magnifications: (d) 5000×, (e) 10 000×, and (f) 20 000×.

SEM has been used to reveal the possible effects of processing on the final thickness of the resulting samples and on the agglomeration/dispersion state of the magnetic nanoparticles within the polymer matrix (Figure 2).

Figure 2a–c displays characteristic SEM images of the samples with 20 wt % CFO processed using three different solvents (a: DMPU, b: DMSO, and c: TEP). No substantial differences are detected between the different composites, all revealing a suitable distribution of cluster of nanoparticles with a maximum size of 3 μm (Figure 2d–f), compatible with a proper ME response.^54^

### Thermal Characteristics and Degree of Crystallinity

**Figure 3:**
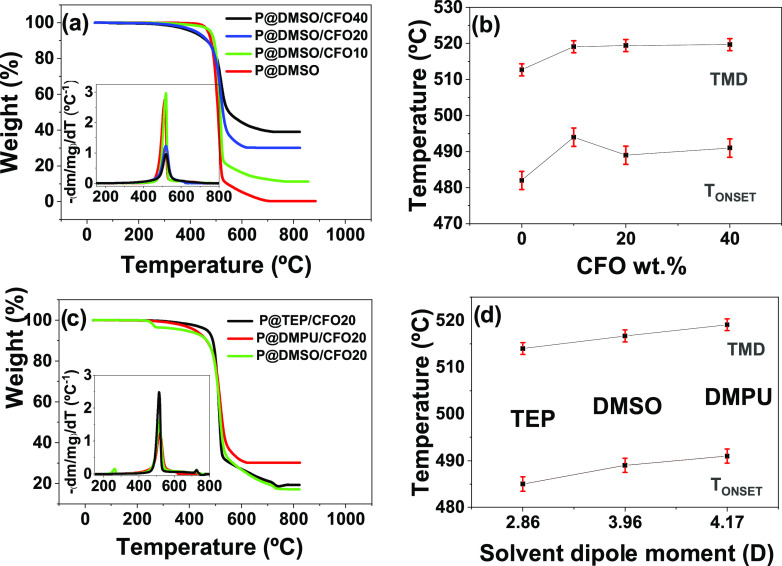
(a, c) TGA thermograms and corresponding first derivatives (insets) for (a) nanocomposites with different CFO wt % contents and (c) nanocomposites prepared with different solvents and under different processing conditions. The temperature of maximum rate of weight loss (TMD) for the samples (b) with different CFO wt % contents and (d) prepared with different solvents and under different processing conditions.

To study the influence of the solvent, processing conditions, and CFO nanoparticle content on the thermal stability of the nanocomposites, TGA has been used, and the representative curves for the different P(VDF-TrFE)/CFO composites are shown in Figure 3.

**Figure 4:**
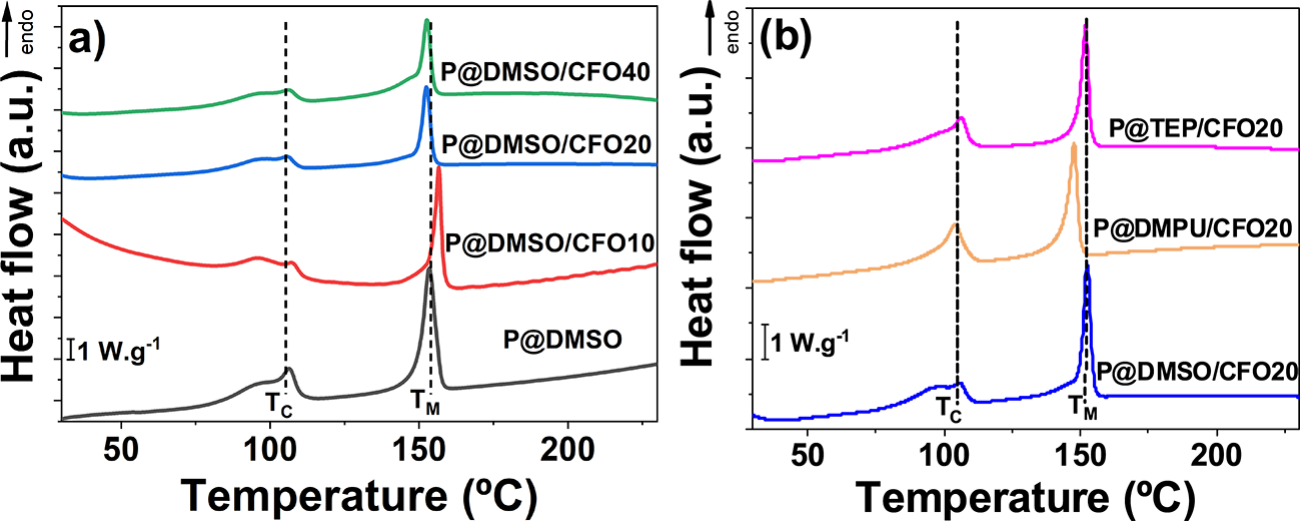
DSC thermograms for samples with different (a) wt % of CFO and (b) different processing conditions and solvents for a filler content of 20 wt % CFO.

It is found that, independently of the CFO wt % content or the solvent and processing conditions, the thermal degradation behavior of P(VDF-TrFE) occurs in one main weight loss step, corresponding to the scission of the carbon–hydrogen (C–H) bonds, followed by the carbon–fluorine (C–F) bonds due to the higher strength of the C–F bonds comparatively to the C–H bonds.^55^Figure 3c compares the composite samples with 20 wt % processed with different solvents, revealing a slight weight loss immediately above the solvents’ evaporation temperature in all samples (also observed in the DTGA curves), which can be related to trapped solvent at the filler–polymer interfaces, and determining also the crystallization (Figure 4) and the morphology of the films (Figure 2), based on the different dipole moments of the solvents. The possibility of the existence of trapped solvent at the filler–polymer interface at crystallization is strengthened by the increased weight loss with increasing CFO wt % (from 0 to ≈2% with increasing wt % from 0 to 40). Additionally, samples with the same CFO wt % (20) but produced with different solvents exhibit the same weight loss (≈0.5%).

The differential thermogravimetric, DTG, analysis (insets of Figure 3a,c) allows us to identify the temperature of maximum rate of weight loss, which is approximately 513 °C for P@DMSO, this value being increased to 519–520 °C with the introduction of CFO nanoparticles. The increase of CFO wt %, from 10 to 40, has no appreciable effect on the TMD. Similarly, the onset temperature is increased from 482 to 489–494 °C with the introduction of CFO nanoparticles. Such behaviors can be related to the emergence of an interphase (≈60% of the sample weight, as calculated using eq 1) in the interface between nanoparticles and polymer resulting from electrostatic interactions among the partially positive C–H bonds of the polymer and the negatively charged CFO (after the solvent trapped at the polymer–particle interface being evaporated), providing larger thermal stability to the polymer chains closer to the ferrite surface.^52,56,57^ The total interface value (≈60%) is similar for P(VDF-TrFE) composites with CFO wt % between 10 and 40 due to the formation of aggregates, variations in the connectivity of the polymer phase, and effects in the internal heat transfer kinetics resulting from the different thermal characteristics of the magnetic nanoparticles and P(VDF-TrFE).^52^

Regarding the effect of the solvent on the thermal properties of the composites, TEP, DMSO, and DMPU led to TMD values of 514, 517, and 519 °C and *T*_ONSET_ values of 485, 489, and 491 °C, respectively, with all variations within experimental error.^35^

Figure 4 shows the heating DSC thermograms for the different P(VDF-TrFE)-based composite films.

The peak at a lower temperature (≈105 °C) corresponds to the ferroelectric–paraelectric transition (Curie transition, *T*_c_), and the higher temperature transition peak at 147–153 °C corresponds to the melting temperature, *T*_m_, of the paraelectric phase.^55^ Regardless of the used solvent or the wt % of CFO, the reference temperatures of the copolymer are not significantly affected (≈100 °C for *T*_C_ and ≈145 °C for *T*_M_).^55,58^

The degree of crystallinity values, calculated from the DSC thermograms by eq 2, are presented in Table 3.

**Table 3:** Effect of Solvent and CFO wt % on the *T*_M_ and *X*_C_ values

solvent	CFO wt %	*T*_M_ (°C) ±1	*X*_c_ (%) ±1
DMSO	0	153	44
	10	156	26
	20	153	25
	40	153	25
DMPU	20	148	31
TEP	20	152	26

Values from Table 3 reveal that the *X*_C_ of the sample without magnetic nanoparticles is ≈40% higher than the ones traditionally reported on P(VDF-TrFE) produced using other solvents (26–32%).^27,55^ Despite previous studies suggesting that the strength of the dipole moment of solvents has a lower impact on the formation of crystals than the annealing process,^59^ for samples with similar annealing, it was reported that a solvent with a higher dipole moment such as DMSO contributes to an improved dipole alignment in P(VDF-TrFE) that results in an augmented *X*_C_.^36,60^ The same effect is also seen on samples prepared with 20 wt % CFO in different solvents, with the degree of crystallinity larger for the samples prepared with DMPU, the solvent with the highest dipole moment.^36,60^

### Mechanical Characteristics

**Figure 5:**
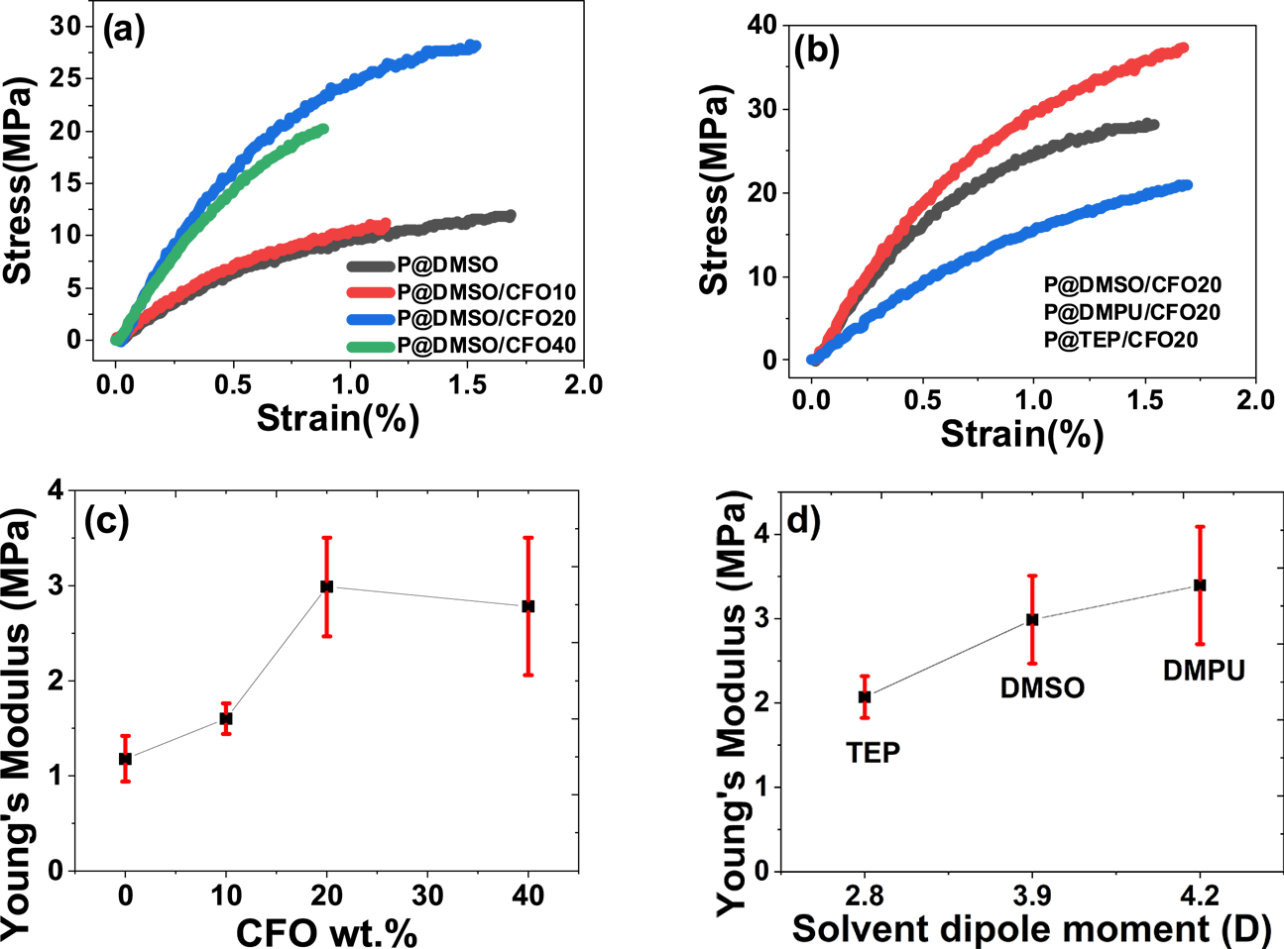
Stress–strain characteristic curves obtained for composites with varying (a) wt % content of ferrites and (b) processing conditions and solvents. Young’s modulus (*E*_Y_Y) as a function of (c) wt % content of ferrites and (d) solvent dipole moment.

To verify whether the P(VDF-TrFE) mechanical properties were changed by the addition of CFO, processing, or solvent dipole moment, stress–strain measurements (Figure 5a,b) have been performed and the corresponding Young’s modulus was calculated (Figure 5c,d).

Figure 5a,b reveals that all composites exhibit the usual mechanical behavior of thermoplastics, with a linear relation between stress and strain, for strains below 1%, followed by a plastic deformation stage before the sample undergoes fracture.

Figure 5c,d shows that the Young’s modulus increases with the introduction of the CFO fillers, this increase being more significant for composites with a higher CFO wt %, a fact that can be related to the mechanical enhancement resulting from the interaction of CFO nanoparticles and the P(VDF-TrFE) chains and also to the higher *Y*_Y_ of CFO (≈150 GPa).^22,61^ In a similar way a solvent with a high dipole moment creates larger solvent /–polymer chain interactions, leading to improved chain orientation and therefore higher Y values (Figure 5d).^36,62^

### Dielectric Response and Electrical Conductivity

**Figure 6:**
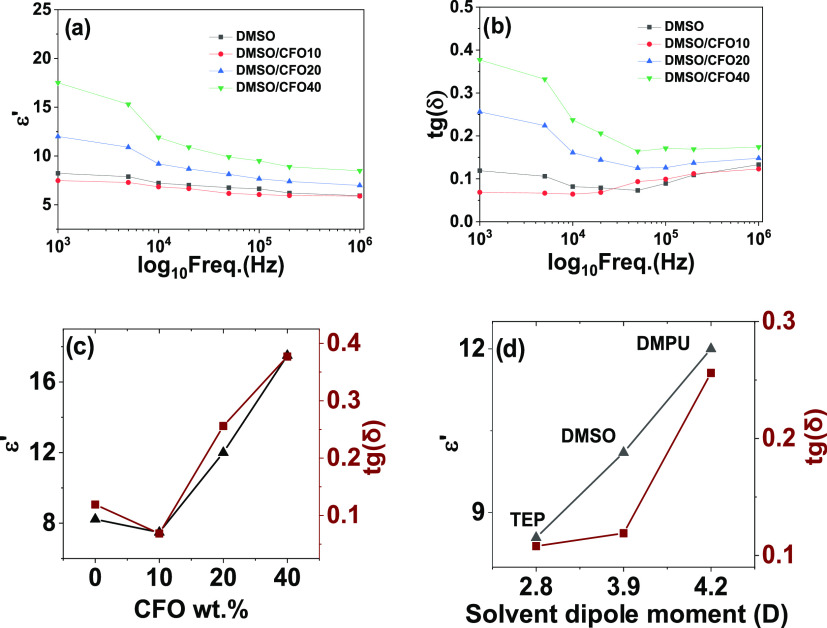
(a) Real part of the dielectric constant and (b) imaginary part of the dielectric response as a function of frequency for P@DMSO composites with different CFO wt %. Variation of the real part of the dielectric function (black triangles) and dielectric losses (red squares) (c) for the P@DMSO nanocomposites as a function of CFO wt % and (d) for the nanocomposites with 20 wt % CFO as a function of solvent dipole moment.

Figure 6 shows the variation of the real part of the dielectric constant, ε′, (Figure 6a) and the dielectric losses, tg(δ), at room temperature (Figure 6b), as a function of the frequency and CFO wt %, for the P(VDF-TrFE)/CFO composites.

For all composite films (independently of the CFO wt %), it is noted that ε′ decreases with increasing frequency in a similar way (Figure 6a) as a result of the cooperative relaxation of the orientational movement of the dipoles with strong contributions from the crystalline–amorphous interphases.^53,63^ The calculated values of the dielectric constant are characteristics of this polymer.^64^ Also, the dielectric losses decrease as the frequency increases for all composites (Figure 6b) due to the relaxation dynamics of the polymer.^65^ At low frequencies, tg(δ) is superior because of the semicrystalline character of the polymer that leads to interfacial polarization contributions. Figure 6c,d displays the variation of the dielectric constant of the samples with different CFO wt % and different solvents, respectively, for a frequency of 10 kHz. This analysis demonstrates that the dielectric response strongly increases with increasing CFO wt %, due to both the dielectric contribution of the filler and the increased number of polymer–filler interfaces (Figure 6c). The dielectric losses show an analogous tendency as that of the real part of the dielectric constant. Concerning the variation of dielectric response and dielectric loss with different solvents at 10 kHz, the sample with higher ε′ is the one prepared with DMPU (≈12), and the sample prepared with TEP presents a lower value (≈8.5). The high dipole moment of DMPU leads to improved chain orientation and therefore a higher dielectric response (Figure 6d).^36^ Further, as indicated in discussion of Figure 3, the solvent seems to remain in the polymer–filler interfaces, making the solvents with higher dipole moments contribute more to the dielectric response.

**Figure 7:**
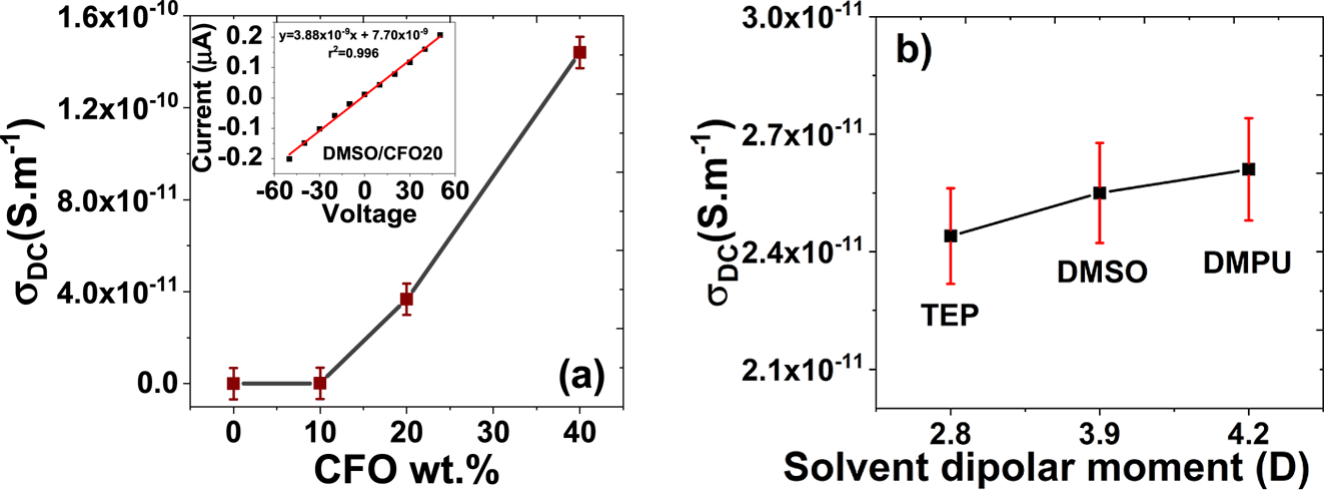
Electrical DC conductivity (σ_DC_) of pristine P(VDF-TrFE) and different nanocomposites as a function of (a) CFO wt % and (b) solvent dipole moment.

The DC conductivity of all composites was evaluated, and the results are shown in Figure 7).

The representative *I*–*V* plots (inset of Figure 7a) reveal a typical ohmic behavior in all P(VDF-TrFE)/CFO composites, with the electric current increasing linearly with the applied voltage. The slope of the *I*–*V* plots and consequently the DC conductivity of the composites increase with the CFO content, from 2.0 × 10^–12^ S m^–1^ for neat P(VDF-TrFE) to 1.4 × 10^–10^ S m^–1^ for the sample with 40 wt % CFO (inset of Figure 7a).

The introduction of magnetic nanoparticles within P(VDF-TrFE) significantly increases its electrical conductivity (Figure 7a), this increase being related to the interfacial charges, the defective interfaces, and the corresponding increase of the conduction paths within the P(VDF-TrFE) matrix as a result of the introduction of the fillers.^66^ Additionally, no substantial effect on the electric conductivity was detected when the solvent was changed (Figure 7b).

For a low CFO content, the composites (less than 40 wt %) show good distribution of CFO nanoparticles in the P(VDF-TrFE) matrix, leading also to high dielectric permittivity (Figure 5), optimized Young’s modulus (Figure 6), and low DC conductivity (Figure 7) that are advantageous for ME device applications.^66,67^

### Piezoelectric and Magnetoelectric Response

**Figure 8:**
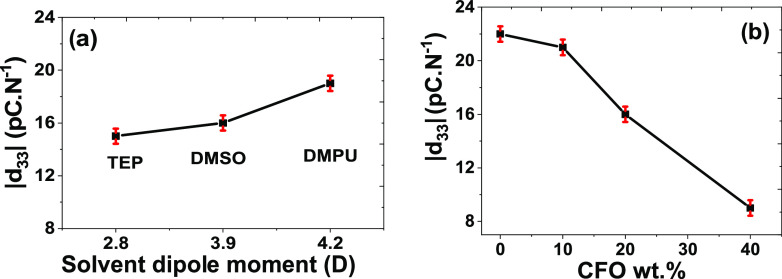
(a) Variation of the *d*_33_ coefficient for the P(VDF-TrFE)/CFO samples with 20 wt % of magnetic nanoparticles as a function of the solvent dipole moment. (b) Variation of the piezoelectric *d*_33_ coefficient for P@DMSO samples as a function of the CFO wt %.

The *d*_33_ piezoelectric coefficient being a key parameter of ME materials, the piezoelectric response of the films has been evaluated as a function of the solvent dipole moment (Figure 8a) and CFO wt % (Figure 8b).

The solvent dipole moment seems to have only a slight effect on the |*d*_33_| values of composites, with the ones found in P@TEP/CFO20 and P@DMSO/CFO20 (15–16 pC N^–1^) increased to 19 pC N^–1^ for the samples prepared with DMPU due to its higher crystallinity (Table 3). Additionally, the presence of the CFO nanoparticles substantially influences the piezoelectric response of the composites. Figure 8b reveals a decrease of |*d*_33_| from 22 to 9 pC N^–1^, with increasing CFO content, caused by a disruption of the connectivity of the piezoelectric matrix, interfacial mechanical defects, increased stiffness of the sample, and reduced crystallinity.^66^

**Figure 9:**
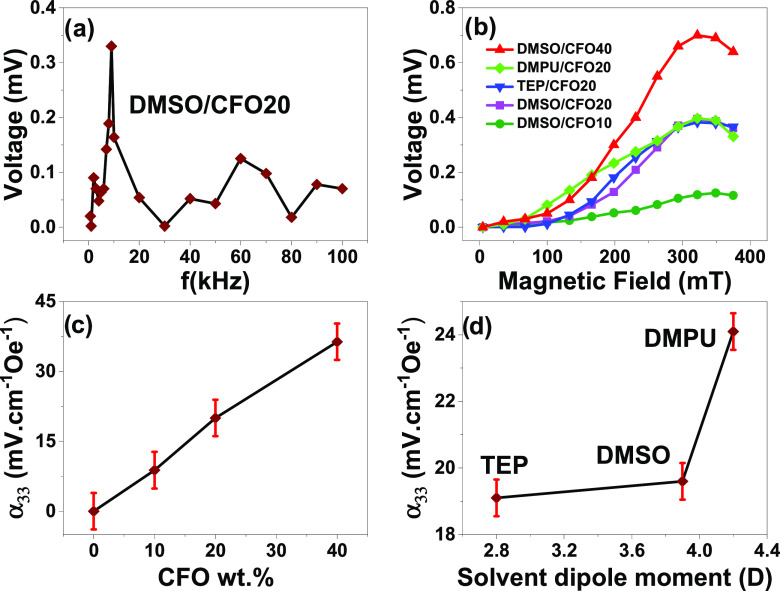
ME voltage as a function of (a) frequency and (b) DC magnetic field intensity. ME voltage coefficient (α_33_) as a function of the CFO wt % content (c) and solvent dipole moment (d).

Finally, the ME properties have been studied as a function of frequency, DC magnetic field intensity, CFO wt % content, and sample preparation method (Figure 9).

Figure 9a shows a typical voltage–frequency plot that reveals that the resonance frequency of the P@DMSO/CFO20 sample is ≈9 kHz. The harmonic mode, thickness of the composites, in-plane Youngʼs modulus, and volumetric mass density of the composites^22^ set the resonance of all P(VDF-TrFE)/CFO composites in the range of 7–9 kHz, with the subsequent characterization being performed at different frequencies (8, 8, 9, and 7 kHz for the P@DMSO/CFO10, P@DMSO/CFO40, P@TEP/CFO20, and P@DMPU/CFO20 composites, respectively).

In all composites, the ME voltage increases with increasing magnetic field, until the optimum magnetic field is reached (≈330 mT) as a result of the higher striction at such a magnetic field (Figure 9b). The following decrease of the ME voltage can be explained by the saturation of the magnetostrictive effect on the magnetostrictive CFO nanoparticles.^21,22^

After the calculation of the ME coefficient (α), it is observed that α increases with increasing CFO content on the P@DMSO/CFO composites (Figure 9c) and with increasing solvent dipole moment on the samples with 20 wt % CFO (Figure 9d). By increasing the CFO content from 10 to 40 wt %, the α value also increases, almost linearly, from 8.8 to 36.4 mV cm^–1^ Oe^–1^, because of the increase in the magnetostriction due to the increase of the magnetostrictive phase on the ME composite. A further increase in the CFO wt % should decrease the α value due to the partial disruption of the piezoelectric phase.^54^ For the same CFO wt % content, α is proportional to the |*d*_33_| piezoelectric coefficient and Young’s modulus values.^68^ The composite that has both of those values optimized P@DMPU/CFO20) also has the highest ME response, as shown in Figure 9d (24 mV cm^–1^ Oe^–1^). Additionally, the sample with the highest CFO wt % P@DMSO/CFO40 exhibited the highest α (35 mV cm^–1^ Oe^–1^) among the studied samples due to the higher content of the magnetostrictive phase and the highest sensitivity reported in the literature for nanocomposites with a similar composition (Table 4).

**Table 4:** Comparison of the ME Response of P(VDF-TrFE)/CFO Composites Exhibiting the Highest ME Coupling Reported in the Literature

CFO (wt %)	*H*_DC_ (T)	thickness (μm)	*H*_AC_ (Oe)	α (mV cm^–1^ Oe^–1^)	*V* (μV)	*S*_DC_ (μV T^–1^)	ref
66	0.25	50	0.008	40	1.6	8	(61)
72	0.25	80	0.1	42	320	1280	(54)
40	0.32	50	3.98	35	700	2188	our

It is shown that although α is similar to the ones reported in the literature for samples prepared with solvents with environmental concerns, the sensitivity, one of the key parameters for the development of ME materials,^23,26^ was optimized under specific applied field conditions for a more effective implementation of these materials in sensing applications.

## Conclusions

This study, in addition to showing that it is possible to prepare ME composites using green solvents, also reveals how some properties of the resulting ME composite such as the maximum degradation temperature, dielectric constant, Young’s modulus, piezoelectric coefficient, and ME voltage coefficient depend on the processing conditions, including solvent selection. The magnetoelectric coupling is similar to the ones reported in the literature for samples prepared with environmentally problematic solvents, and the sensitivity of the proposed ME nanocomposites under specific *H*_AC_ conditions (2188 μV T^–1^) is almost double the highest value reported in the literature (1280 μV T^–1^).
